# Eintreffzeiten des Rettungsdienstes bei außerklinischem Herz-Kreislauf-Stillstand – überlebensrelevante Unterschiede zwischen den Bundesländern in Deutschland

**DOI:** 10.1007/s00101-025-01592-9

**Published:** 2025-09-16

**Authors:** Matthias Fischer, Ulf Harding, Harald Genzwürker, Stephan Seewald, Peter Gretenkort, Florian Reifferscheid

**Affiliations:** 1 Arbeitsgemeinschaft Südwestdeutscher Notärztinnen und Notärzte e. V. (AGSWN), Filderstadt, Deutschland; 2 DRK Göppingen, Göppingen, Deutschland; 3 IRUN, Universitätsklinikum Schleswig-Holstein, Campus Kiel, Kiel, Deutschland; 4 Arbeitsgemeinschaft in Norddeutschland tätiger Notärztinnen und Notärzte e. V. (AGNN), Lübeck, Deutschland; 5 Zentrum für Notfallmedizin, Städtisches Klinikum Wolfenbüttel gGmbH, Wolfenbüttel, Deutschland; 6 Leitende Notarztgruppe Neckar-Odenwald-Kreis, Buchen, Deutschland; 7 Klinik für Anästhesiologie und Operative Intensivmedizin, Universitätsklinikum Schleswig-Holstein, Campus Kiel, Arnold-Heller-Straße 3, Haus R3, 24105 Kiel, Deutschland; 8 Bundesvereinigung der Arbeitsgemeinschaften Notärzte Deutschlands (BAND), Berlin, Deutschland; 9 Deutsche Gesellschaft für Notfallmedizin e. V. (DGfNM), Berlin, Deutschland; 10 Simulations- und Notfallakademie, HELIOS Klinikum Krefeld, Krefeld, Deutschland

**Keywords:** Hilfsfrist, Rettungsdienstplanung, Reanimation, Überleben, Rettungsdienst, Response time, Emergency Service Planning, Resuscitation, Survival, Emergency medical service

## Abstract

**Hintergrund:**

Die Hilfsfrist ist eine wichtige Planungsgröße; sie bedingt die Eintreffzeiten des Rettungsdienstes und wird in Ländergesetzen geregelt. Diese Studie analysiert die Eintreffzeiten in den Bundesländern.

**Methode:**

Im Deutschen Reanimationsregister werden die Eintreffzeiten für das ersteintreffende Fahrzeug und das vollständige Team (Team-Eintreffzeit: Rettungswagen und Notarzt) bundeseinheitlich erfasst. Die statistische Prüfung der Nullhypothese, Gleichheit zwischen den Bundesländern, erfolgte mit dem Kruskal-Wallis-Test. Mittels binär logistischen Regressionsanalysen wurde der Einfluss von Eintreffzeiten auf die Überlebensraten untersucht.

**Ergebnisse:**

Die Null-Hypothese musste verworfen werden (*p* < 0,001); die Eintreffzeiten sind in den Bundesländern ungleich (Mittelwerte für die Eintreffzeit: 5,7 ± 2,6 min bis 7,4 ± 3,5 min, 90. Perzentile 9–13 min; Mittelwerte der Team-Eintreffzeit: 8,6 ± 3,6 min bis 11,9 ± 5,9 min; 90. Perzentile 13–21 min; 2014–2024: *n* = 104.657; 201 Rettungsdienste). In 10 von 16 Bundesländern konnten jeweils weniger als 80 % der Patienten innerhalb von 8 min vom ersten Fahrzeug erreicht werden.

Die Regressionsanalysen zeigten, dass zunehmende Eintreff- und Team-Eintreffzeiten mit guter neurologischer Erholung bei Entlassung negativ assoziiert sind (Team-Eintreffzeit ≥ 12 min: OR =0,54, KI =0,39–0,75, *p* < 0,001; *n* = 45.873; 71 Referenzstandorte).

**Schlussfolgerung:**

Patienten erhalten in den verschiedenen Bundesländern eine qualitativ unterschiedliche Versorgung, da sich Eintreffzeiten unterscheiden. Kürzere Eintreffzeiten sind mit besseren Überlebenschancen assoziiert. Eine gleiche Versorgungsqualität ist grundgesetzlich gefordert, jedoch nicht umgesetzt.

**Graphic abstract:**

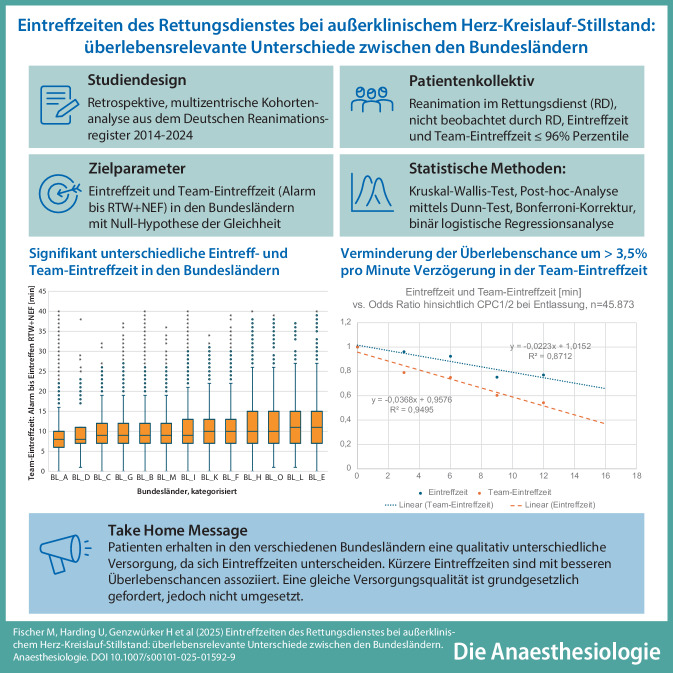

## Abstract (en)

**Background:**

The response time (“Hilfsfrist”) in Germany is an important planning parameter that determines the arrival times of emergency medical services and is regulated by state legislation. This study analyzes arrival times in the German federal states.

**Material and method:**

The German Resuscitation Registry uniformly records arrival times for the first vehicle on the scene and the complete team (team arrival time: ambulance and emergency physician) nationwide. The statistical testing of the null hypothesis, equality between the federal states, was conducted using the Kruskal-Wallis test. Binary logistic regression analyses were used to examine the impact of arrival times on survival rates.

**Results:**

The null hypothesis had to be rejected (*p* < 0.001), indicating that arrival times differ between the federal states (first vehicle: mean arrival times ranged from 5.7 ± 2.6 min to 7.4 ± 3.5 min; 90th percentile: 9–13 min; team arrival time ranged from 8.6 ± 3.6 min to 11.9 ± 5.9 min; 90th percentile: 13–21 min; 2014–2024: *n* = 104,657; 201 ambulance services). In 10 out of 16 federal states, fewer than 80% of patients were reached by the first vehicle within 8 min.

The regression analyses showed that longer arrival and team arrival times were negatively associated with good neurological recovery at discharge (team arrival time ≥ 12 min: odds ratio, OR =0.54, confidence interval, CI =0.39–0.75, *p* < 0.001; *n* = 45,873; 71 reference emergency medical services, EMS, sites).

**Conclusion:**

Patients receive qualitatively different levels of care across the federal states due to variations in arrival times. Shorter arrival times are associated with better chances of survival. Equal quality of care is a constitutional requirement by the German constitution but it is not being achieved.

## Einleitung

Der Behandlungserfolg bei außerklinischem Herz-Kreislauf-Stillstand (Out-of-Hospital Cardiac Arrest, OHCA) ist in hohem Maße zeitabhängig. Studien aus Schweden, Dänemark, England und Deutschland zeigten bei Patienten mit OHCA einen signifikanten Zusammenhang zwischen der Eintreffzeit des ersten Rettungsmittels am Einsatzort und der Häufigkeit der Wiederherstellung eines Spontankreislaufs (Return of Spontaneous Circulation, ROSC) bis zur Klinikaufnahme einerseits sowie einer Entlassung aus dem Krankenhaus andererseits [7, 20, 24, 27].

Zudem zeigte eine Studie aus Deutschland, dass in Rettungsdiensten mit kürzeren Eintreffzeiten mehr Patienten reanimiert werden und mehr Patienten lebend aus dem Krankenhaus entlassen wurden, jeweils bezogen auf 100.000 Einwohnende und Jahr (höhere Reanimations- und Entlassungsinzidenz) [7].

Die Versorgung von OHCA-Patienten erfolgt in Deutschland im Rendezvous-System eines Rettungswagens zusammen mit einem arztbesetzten Rettungsmittel (Notarzteinsatzfahrzeug, NEF; Rettungshubschrauber, RTH). Darüber hinaus können lokal noch weitere Kräfte (z. B. aktivierte Ersthelfer, First Responder, Fahrzeuge der Feuerwehr) hinzukommen, welche aber nicht regelhaft dem Rettungsdienst zugeordnet werden, sowie in Ausbildung und Ausstattung nicht vergleichbar sind und deswegen die Hilfsfrist nicht markieren können.

Für die Gesundheitsversorgung und damit auch für den Rettungsdienst leitet sich aus dem Grundgesetz der Bundesrepublik Deutschland ein infrastruktureller Mindeststandard als Aufgabe der staatlichen Daseinsvorsorge ab. Allerdings haben die Bundesländer in ihrer Gesetzgebung einen weiten Gestaltungsspielraum bei der Festlegung von Leistungsansprüchen zu einer ausreichenden medizinischen Versorgung [5]. Die Bedarfsplanung von Standorten und Fahrzeugen des Rettungsdienstes erfolgt unter Berücksichtigung sog. planerischer Hilfsfristen. Die Bundesländer verwenden unterschiedliche planerische Hilfsfristen in den Landesrettungsdienstgesetzten (Tab. 1).

**Tab. 1:** Planerische Hilfsfristen, Einwohnerzahlen und Bevölkerungsdichte in den Bundesländern. (Nach Di Fabio [8]). Es ist zu beachten, dass Baden-Württemberg im Jahr 2024 die notärztliche Hilfsfrist abgeschafft und die Eintreffzeit mit ≤ 12 min und 95 %igem Erreichungsgrad ab Einsatzannahmeende definiert hat.

Bundesland	Baden-Württemberg	Bayern	Berlin	Brandenburg	Bremen	Hamburg	Hessen	Mecklenburg-Vorpommern	Niedersachsen	Nordrhein-Westfalen	RHeiland-Pfalz	Saarland	Sachsen	Sachsen-Anhalt	Schleswig-Holstein	Thüringen
Einwohner (Mio.) *	11,3	13,4	3,8	2,6	0,7	1,9	6,4	1,6	8,1	18,1	4,2	1	4,1	2,2	3	2,1
Bevölkerungsdichte (E./km^2^) *	316	190	4214	87	1632	2506	303	70	171	532	209	386	221	107	187	131
*Hilfsfrist (min)*																
Allgemein	**Mögl. 10, max. 15**	**12**	**8**	**15**	**10**	**„flächendeckende, bedarfs- und fachgerechte Versorgung“**	**10**	**10**	**15**	**8 (Stadt)**	**15 (Fahrzeit)**	**12**	**12**	**12**	**12**	**12**
Land	**–**	**–**	**–**	**–**	**–**	**–**	**–**	**–**	**–**	**12**	**–**	**–**	**–**	**–**	**(-) geogr. erschwert zugänglicher Ort**	**15**
Notarzt	**s.** **oben**	**–**	**–**	**–**	**–**	**–**	**–**	**–**	**–**	**–**	**–**	**–**	**–**	**20**	**–**	**–**
Perzentil (planer. Erreichungsgrad)	**95**	**–**	**75**	**95**	**95**	**–**	**–**	**–**	**95**	**90**	**–**	**95**	**95**	**95**	**90**	**–**
Fristbeginn	Eingang Meldung	Ausrücken	Eingang Meldung	Eingang Meldung	Eröffnung Einsatz	–	Eingang	Alarmierung	Einsatzentscheidung	Disposition	Eingang Meldung	Einsatzentscheidung	–	Eingang Meldung	Alarmierung	–
Grundlage	§ 3 II RDG, § 6 RDPlan (2022)	§ 2 I AV-BayRDG 80 % Notfallereignisse gem. Erlass	Vereinbarung Senat + Feuerwehr	§ 8 BdgRettG	§ 28 II Brem-HilfeG	§ 12 I HmbRDG	§ 15 Abs. 2 Satz 2 HRDG	§ 8 II Nr. 7 RDG-MV	§ 2 III BedarfVO RettG	Nur Empfehlungen	§ 8 II RettDG	§ 6 Abs. 3 SRettG	§ 26 II Säch-BRKG, § 4 I SächLRettDPVO	§ 2 XVII, § 7 IV RettDG LSA	§ 4 II SHRDG, § 2 I, II SHRDG-DVO	§ 12 I Nr. 1 ThürRettG

* Daten für 2022 aus: Statistische Ämter des Bundes und der Länder, https://www.statistikportal.de/de/bevoelkerung/flaeche-und-bevoelkerung

Die Eintreffzeit des Rettungsdienstes wird in der elektronischen Einsatzdokumentation zu dem Zeitpunkt dokumentiert, an dem das Fahrzeug den Einsatzort erreicht. In der digitalen Funkalarmierung gemäß den *Technischen Richtlinien der Behörden und Organisationen mit Sicherheitsaufgaben (TR BOS)* wird das mit Eingabe des „Status 4“ bestätigt.

Eintreff- und Prozesszeiten nach OHCA werden im Deutschen Reanimationsregister (German Resuscitation Registry, GRR) bundeseinheitlich erfasst [15]. Das GRR ist das größte rettungsdienstliche Notfallregister in Deutschland und ermöglicht ein sektorenübergreifendes Qualitätsmanagement [12]. Über die Eintreffzeiten hinaus werden im GRR umfangreiche Parameter gemäß der Utstein-Definitionen [14], die für die Prozessanalyse, Auswertung, Risikoadjustierung und Ergebnisanalyse von Reanimationen von Bedeutung sind, erfasst. Die Datenerhebung erfolgt anhand eines Erfassungsbogens, über eine webbasierte Eingabemaske oder durch einen Datenimport aus dem digitalen Dokumentationssystem des jeweiligen Rettungsdienstes in das GRR [15].

## Fragestellung

Anhand aktueller Daten des Reanimationsregisters wird die Nullhypothese überprüft, ob die Bürgerinnen und Bürger im Falle eines OHCA in allen Bundesländern eine gleich schnelle medizinische Versorgung durch Notarzt- und Rettungsdienst erhalten.

Neben der Analyse der Eintreffzeiten des ersten Fahrzeugs (RTW oder NEF/RTH) sollen die Eintreffzeiten des zweiten Fahrzeugs, die wir hier als Team-Eintreffzeiten definieren, betrachtet werden. Mit dem zweiten Fahrzeug wird die taktische Einheit aus Rettungswagen und arztbesetztem Rettungsmittel vervollständigt, sodass leitliniengerechte Maßnahmen zur Reanimation effektiver umgesetzt werden können [10]. „First Responder“ oder „aktivierte Ersthelfende“, welche als „Vorausversorger“ neben dem regulären Rettungsdienst alarmiert wurden, werden bei den oben genannten Eintreffzeiten nicht berücksichtigt.

Im zweiten Teil wird untersucht, ob und in welchem Ausmaß Eintreffzeiten und Team-Eintreffzeiten das Überleben nach Herz-Kreislauf-Stillstand beeinflussen.

## Methodik

Diese retrospektive Registerstudie erfolgt anhand anonymisierter Daten des GRR des Zeitraums 01.01.2014 bis 31.12.2024. Positive Bewertungen durch die Ethikkommission der Medizinischen Fakultät der Christian-Albrechts-Universität zu Kiel (D 645/24) sowie des Wissenschaftlichen Beirats des GRR (2025-01) liegen vor. Bei der Manuskripterstellung wurden die Deklaration von Helsinki und die Publikationsrichtline des GRR (Version 6/2024) berücksichtigt.

In die Studie wurden Patienten mit OHCA und Reanimation eingeschlossen, wenn die beteiligten Standorte in Deutschland über mindesten ein Jahr im Untersuchungszeitraum von 11 Jahren Daten übermittelt hatten. Das GRR ist ein Register auf der Basis einer freiwilligen Teilnahme, es verzeichnet eine stetig zunehmende Zahl von Teilnehmern, von denen aber nur ein Teil seit 2014 ununterbrochen Daten geliefert hat. Ausgeschlossen wurden Fälle, bei denen der OHCA im Beisein des Rettungsdienstes eintrat oder deren Eintreffzeiten oder Team-Eintreffzeiten nicht dokumentiert wurden. 4233 Datensätze wurden zudem wegen Implausibilität (> 96 % Perzentile) ausgeschlossen, wenn die Eintreffzeit 30 min oder die Teameintreffzeit 40 min überschritt.

Im ersten Teil der Studie wurden Eintreffzeiten und Team-Eintreffzeiten sowie andere Prozesszeiten aller beteiligten Rettungsdienste, stratifiziert nach den einzelnen Bundesländern, untersucht. Die statistische Prüfung der Nullhypothese (Gleichheit zwischen den Bundesländern) erfolgte mit dem Kruskal-Wallis-Test zum Signifikanzniveau *p* < 0,05 sowie einer Post-hoc-Analyse mittels Dunn-Test und einer Bonferroni-Korrektur für multiples Testen. Die Bundesländer wurden dabei zur Einhaltung der Anonymisierung unter den zufällig vergebenen Buchstaben A–O aufgelistet. Die Stadtstaaten Berlin, Bremen und Hamburg sowie das Saarland wurden in einer eigenen Gruppe zusammengefasst. Eine Deanonymisierung der Bundesländer ist entsprechend den Verträgen zwischen Teilnehmern und GRR nur möglich, wenn alle Teilnehmer dieser Offenlegung zustimmen.

Im zweiten Teil der Studie wurde der Einfluss der Eintreffzeit und der Team-Eintreffzeit auf die abhängigen Variablen ROSC bei Klinikaufnahme, Entlassung aus der Klinik bzw. 30-Tage-Überleben sowie Überleben mit guter neurologischer Erholung (*Cerebral Performance Categories*, CPC 1 oder 2) mittels binär logistischer Regressionsanalysen untersucht. In die Regressionsanalysen wurden mögliche Einflussvariablen bei *p* < 0,05 eingeschlossen. Für die eingeschlossenen Variablen wurden Regressionskoeffizienten, Odds Ratios und Konfidenzintervalle errechnet und ein Forest Plot erstellt. Die untersuchten unabhängigen Einflussvariablen basieren auf dem *ROSC after Cardiac Arrest Score (RACA)* [13, 21] und dem *CaRdiac Arrest Survival Score (CRASS)* [29] und sind entsprechend als kategoriale Variablen angelegt sowie im Detail in der Infobox definiert und gelistet. Die Eintreff- und Team-Eintreffzeit wurden in 3 min-Gruppen eingeteilt, um die Vergleichbarkeit zur bestehenden Literatur herzustellen.

Bei der Betrachtung zum Endpunkt „Krankenhausaufnahme mit ROSC“ wurden die Daten aller Rettungsdienste eingeschlossen. In die Analysen zu den Endpunkten der klinischen Versorgung wurden nur die Daten der Referenzstandorte, welche im GRR wie folgt definiert sind, eingeschlossen [10]:

- Inzidenz für Reanimationen > 30/100.000 Einwohnende und Jahr,
- Häufigkeit von jemals ROSC < 80 %,
- Berechenbarkeit des RACA in > 60 % der Fälle möglich,
- Anteil an dokumentierten klinischen Weiterversorgungen > 30 %.

Die Datenverarbeitung erfolgte mittels Microsoft® Excel® für Microsoft 365 MSO (Version 2312 Build 16.0.17126.20132, 64 Bit) sowie IBM SPSS Statistics Version 29.0.2.0 (20) (IBM, Armonk, NY, USA).

## Ergebnisse

Im untersuchten Zeitraum 2014–2024 beteiligten sich insgesamt 201 Rettungsdienststandorte aus allen Bundesländern am GRR [10, 11]. Für diese Studie standen entsprechend den Einschluss- und Ausschlusskriterien die Daten von 104.657 Patienten zur Verfügung. Aus der Untergruppe von 71 Referenzstandorten wurden 46.914 Falldatensätze beigetragen.

### Eintreffzeiten und Team-Eintreffzeiten im Vergleich der Bundesländer

**Abb. 1:**
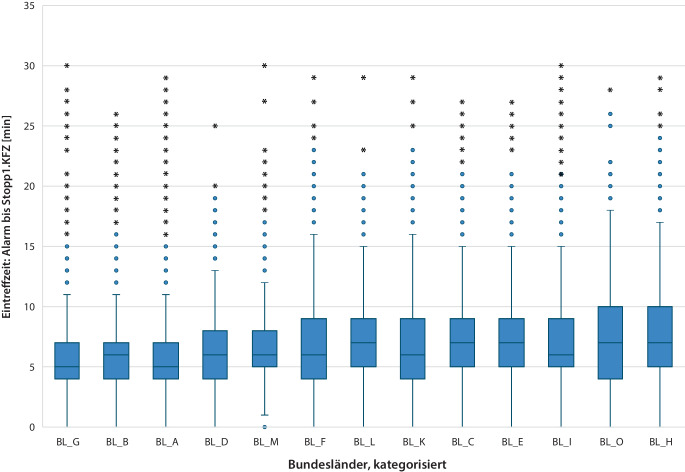
Gegenüberstellung der Eintreffzeiten in den einzelnen Bundesländern für das 1. Fahrzeug (*n* = 104.657; modifizierter Boxplots für Median und Interquartilbereich sowie Gesamtbereich mit Ausreißern begrenzt auf 30 min)

Eintreff‑, Team-Eintreff- und Prozesszeiten für wesentliche therapeutische Maßnahmen im Vergleich der Bundesländer sind in Tab. 2 deskriptiv aufgelistet. In Abb. 1 und 2 sind die Eintreffzeiten bzw. Team-Eintreffzeiten nach Bundesländern grafisch als Boxplots dargestellt. Der statistische Vergleich mit dem Kruskal-Wallis-Test führt in beiden Fällen dazu, dass die Null-Hypothese verworfen werden muss: Die Eintreffzeiten und Team-Eintreffzeiten sind in den Bundesländern nicht gleich, sondern differieren signifikant mit Medianwerten zwischen 5 und 7 min (Eintreffzeit 1. Fahrzeug) bzw. 8 und 11 min (Team-Eintreffzeit). Die Werte für die 90. Perzentile differieren zwischen 9 und 13 min (Eintreffzeit 1. Fahrzeug) bzw. 13 und 20 min (Team-Eintreffzeit). In 10 von 16 Bundesländern werden innerhalb von 8 min jeweils weniger als 80 % vom ersten Fahrzeug nach Alarmierung erreicht, in 3 Bundesländern werden sogar weniger als 65 % der OHCA-Patienten innerhalb von 8 min erreicht (Infobox).

**Tab. 2:** Gegenüberstellung der Eintreffzeiten und weiterer Prozesszeiten in den einzelnen Bundesländern (Mittelwerte und Standardabweichungen).

Bundesland	Anzahl Patienten	Eintreffzeit Alarm bis Eintreffen 1. Fahrzeug [min]	Alarm bis Eintreffen RTW [min]	Patienten, die in 8 min vom 1. Fahrzeug erreicht werden [%]	Alarm bis Eintreffen NEF [min]	Team-Eintreffzeit Alarm bis Eintreffen RTW und NEF [min]	Alarm bis Beatmung [min]	Alarm bis ITN [min]	Alarm bis IVZ [min]	Alarm bis 1. Defibrillation [min]	Alarm bis 1. Defibrillation bei VF/VT [min]	Alarm bis Adrenalin [min]	Alarm bis Adrenalin bei nicht VF/VT [min]	Alarm bis ROSC [min]	Alarm bis Übergabe [min]
Deutschland	104.693	6,2 ± 3,2	6,7 ± 3,7	75,7	9,2 ± 4,5	9,6 ± 4,6	9,3 ± 4,8	18,7 ± 13,6	14,5 ± 5,4	14,2 ± 12,1	10,2 ± 8,5	15,4 ± 8,6	15,3 ± 8,4	23,9 ± 13,0	62,6 ± 20,5
BL_A	34.420	5,7 ± 2,6	6,1 ± 3,1	83,3	8,2 ± 3,7	8,6 ± 3,8	8,8 ± 4,3	18,1 ± 13,6	14,0 ± 5,0	13,8 ± 11,2	9,6 ± 7,0	14,9 ± 8,1	14,7 ± 7,9	23,3 ± 12,4	60,5 ± 18,8
BL_B	13.848	5,9 ± 2,9	6,5 ± 3,7	80,2	8,9 ± 4,3	9,5 ± 4,5	9,5 ± 4,7	17,4 ± 10,9	14,9 ± 5,4	13,7 ± 11,4	9,7 ± 8,0	15,5 ± 7,7	15,4 ± 7,5	23,9 ± 12,7	62,5 ± 20,3
BL_C	4178	7,4 ± 3,5	7,9 ± 3,9	60,4	9,0 ± 3,9	9,4 ± 4,1	9,2 ± 5,2	23,4 ± 22,2	14,2 ± 5,6	17,2 ± 19,7	13,2 ± 18,0	16,0 ± 10,3	15,8 ± 9,7	24,2 ± 14,1	65,1 ± 21,0
BL_D	1896	6,1 ± 3,0	6,7 ± 3,6	76,3	8,4 ± 3,7	9,0 ± 3,8	9,9 ± 4,7	16,8 ± 9,3	15,2 ± 5,0	13,6 ± 9,4	9,5 ± 5,5	15,1 ± 6,2	15,1 ± 6,1	23,5 ± 12,1	58,7 ± 18,0
BL_E	2011	7,3 ± 3,9	7,8 ± 4,4	64,2	11,4 ± 5,9	11,9 ± 5,9	9,7 ± 5,0	18,7 ± 13,5	13,2 ± 5,3	14,8 ± 13,1	10,4 ± 7,0	15,1 ± 11,1	14,7 ± 11,2	26,1 ± 14,0	66,9 ± 23,5
BL_F	8225	6,8 ± 3,6	7,1 ± 3,9	68,7	10,1 ± 5,0	10,4 ± 5,0	9,6 ± 4,9	18,8 ± 10,1	15,1 ± 5,7	13,7 ± 10,1	10,0 ± 6,3	15,7 ± 7,2	15,6 ± 7,3	23,2 ± 12,0	63,6 ± 20,3
BL_G	14.355	5,8 ± 2,9	6,2 ± 3,3	80,0	9,6 ± 4,6	10,0 ± 4,6	9,2 ± 4,5	18,8 ± 12,2	14,9 ± 5,4	14,1 ± 12,0	10,0 ± 6,9	15,9 ± 9,1	15,8 ± 9,0	24,2 ± 14,0	68,6 ± 22,2
BL_H	4220	7,3 ± 4,1	8,1 ± 4,9	63,2	10,5 ± 5,7	11,3 ± 5,9	11,3 ± 5,7	20,7 ± 13,6	15,3 ± 6,1	16,1 ± 14,0	12,9 ± 12,4	17,0 ± 12,1	16,9 ± 12,2	27,1 ± 15,9	68,2 ± 25,5
BL_I	12.650	6,9 ± 3,5	7,3 ± 3,8	66,6	9,7 ± 4,7	10,1 ± 4,7	9,6 ± 5,3	19,1 ± 15,0	14,9 ± 5,5	14,6 ± 12,7	11,0 ± 9,6	15,5 ± 8,6	15,3 ± 8,2	23,9 ± 12,9	60,3 ± 20,0
BL_K	2882	6,6 ± 3,8	6,9 ± 4,2	67,7	9,9 ± 4,7	10,3 ± 4,8	9,3 ± 5,2	18,8 ± 13,5	15,2 ± 5,6	13,8 ± 11,4	9,6 ± 7,5	15,9 ± 8,3	15,6 ± 8,2	24,5 ± 14,1	64,0 ± 21,1
BL_L	1487	7,2 ± 3,8	7,6 ± 4,0	62,3	11,3 ± 5,8	11,6 ± 5,7	9,3 ± 4,7	17,1 ± 7,1	13,1 ± 5,1	14,5 ± 9,3	10,7 ± 5,8	13,0 ± 5,6	12,8 ± 5,5	23,7 ± 12,2	62,2 ± 22,2
BL_M	2933	6,5 ± 3,2	7,0 ± 3,5	73,2	9,3 ± 4,5	9,7 ± 4,4	11,8 ± 5,4	19,9 ± 13,6	15,5 ± 5,1	16,5 ± 12,4	11,6 ± 9,5	17,8 ± 13,8	17,3 ± 13,0	27,0 ± 15,0	57,4 ± 17,1
BL_O	1588	7,4 ± 4,1	8,0 ± 4,5	61,5	10,8 ± 5,9	11,5 ± 5,9	10,5 ± 5,5	17,2 ± 9,5	15,2 ± 5,7	15,3 ± 11,9	11,7 ± 11,0	15,2 ± 7,3	14,8 ± 7,5	25,8 ± 13,3	60,6 ± 19,7

Zu beachten ist, dass die Anzahl der Patienten nicht auf die Einwohnerzahl der Bundesländer rückschließen lässt, da die Teilnahmequote der Rettungsdienste je Bundesland unterschiedlich sein kann.

*RTW* Rettungstransportwagen, *NEF* Notarzteinsatzfahrzeug, *ITN* endotracheale Intubation, *IVZ* intravenöser Zugang, *VF/VT* defibrillierbarer Rhythmus, *ROSC* Return of Spontaneous Circulation

**Abb. 2:**
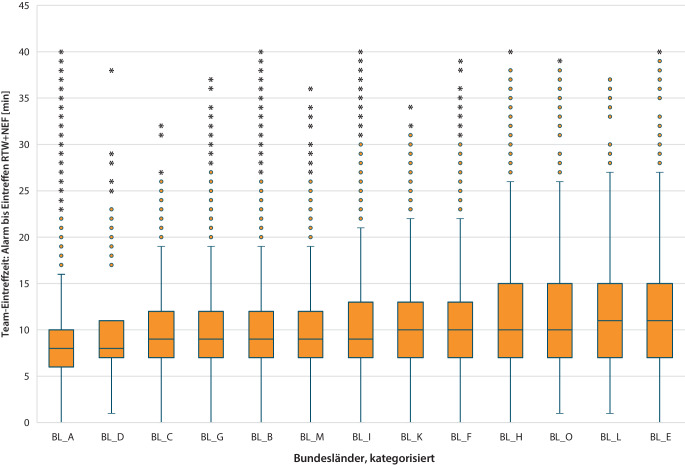
Gegenüberstellung der Team-Eintreffzeiten (Vollständigkeit der taktischen Einheit aus Notarzt und RTW) in den einzelnen Bundesländern (*n* = 104.657; modifizierter Boxplots für Median und Interquartilbereich sowie Gesamtbereich mit Ausreißern begrenzt auf 40 min)

### Auswirkungen von Eintreffzeiten und Team-Eintreffzeiten auf ROSC bei Klinikaufnahme und auf Entlassung mit guter neurologischer Erholung (CPC 1-2)

**Abb. 3:**
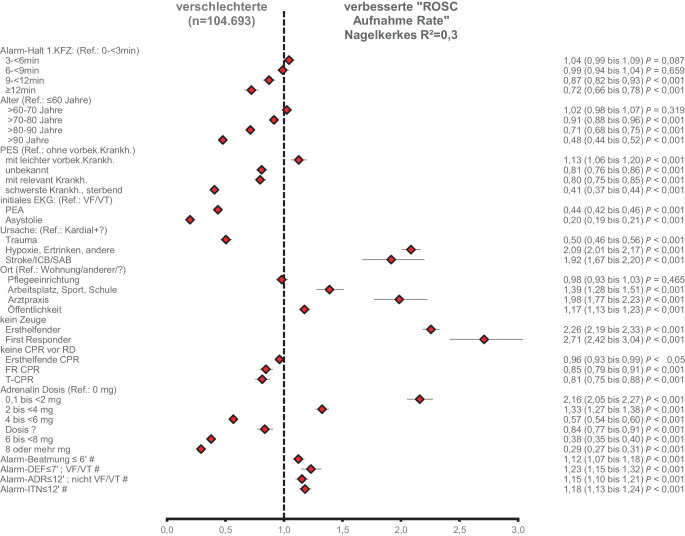
Forest Plot der Odds Ratios mit Konfidenzintervall der multivariaten binären logistischen Regressionsanalyse mit dem Endpunkt „ROSC bei Krankenhausaufnahme“ (*n* = 104.657; Nagelkerkes R^2^ = 0,3). In der *rechten* Spalte sind Odds Ratio (Konfidenzintervall) und *p*-Wert der Regressionsanalyse aufgeführt. *Raute* Referenz alle anderen, *Ref*. Referenzkategorie, *VE* vorbestehende Erkrankung, *PES* Pre-Emergency-Status (Vor-Notfall-Status), *EKG* Elektrokardiogramm, *VF/VT* Kammerflimmern/pulslose ventrikuläre Tachykardie, *PEA* pulslose elektrische Aktivität, *ICB* intrakranielle Blutung, *SAB* Subarachnoidalblutung, *?* unbekannt, *CPR* kardiopulmonale Reanimation, *T‑CPR* telefonisch angeleitete Ersthelfenden CPR, *FR*-*CPR* CPR begonnen durch First Responder, *Alarm-DEF* Zeitintervall Alarm bis zur ersten Defibrillation, *Alarm-Adr* Zeitintervall Alarm bis zur ersten Adrenalingabe, *Alarm-ITN* Zeitintervall Alarm bis zur endotrachealen Intubation

Die Ergebnisse der Regressionsanalysen zu den Endpunkten ROSC bei Klinikaufnahme und -entlassung mit guter neurologischer Erholung (CPC 1‑2) sind in Abb. 3 und 4 als Forest Plots dargestellt. Neben anderen Prozessvariablen haben darin die Eintreffzeiten und Team-Eintreffzeiten in den angegebenen Zeitintervallen einen proportionalen negativen Einfluss auf die Endpunkte. Das Modell der Regression zeigt insbesondere für den Einfluss der Team-Eintreffzeit eine gute Vorhersagekraft (Nagelkerkes R^2^ = 0,446).

**Abb. 4:**
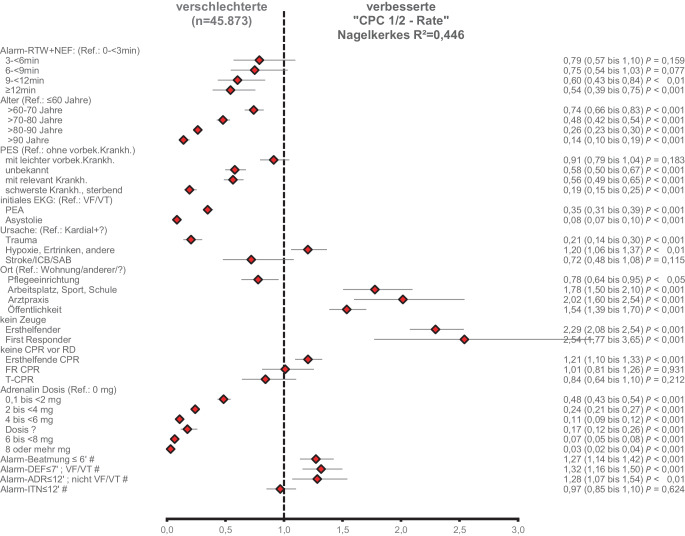
Forest Plot der Odds Ratios mit Konfidenzintervall der multivariaten binären logistischen Regressionsanalyse mit dem Endpunkt „CPC 1‑2 bei Entlassung“ (*n* = 45.873; Nagelkerkes R^2^ = 0,446). In der *rechten *Spalte sind Odds Ratio (Konfidenzintervall) und *p*-Wert der Regressionsanalyse aufgeführt. *Raute* Referenz alle anderen, *Ref*. Referenzkategorie, *VE* vorbestehende Erkrankung, *PES* Pre-Emergency-Status (Vor-Notfall-Status), *EKG* Elektrokardiogramm, *VF/VT* Kammerflimmern/pulslose ventrikuläre Tachykardie, *PEA* pulslose elektrische Aktivität, *ICB* intrakranielle Blutung, *SAB* Subarachnoidalblutung, *?* unbekannt, *CPR* kardiopulmonale Reanimation, *T‑CPR* telefonisch angeleitete Ersthelfenden CPR, *FR-CPR* CPR begonnen durch First Responder, *Alarm-DEF* Zeitintervall Alarm bis zur ersten Defibrillation, *Alarm-Adr* Zeitintervall Alarm bis zur ersten Adrenalingabe, *Alarm-ITN* Zeitintervall Alarm bis zur endotrachealen Intubation

Demzufolge verringert eine zunehmende Team-Eintreffzeit die Chancen auf ein Überleben mit guter neurologischer Erholung nach OHCA und Reanimation signifikant und relevant. Daneben sind u. a. zunehmendes Alter, Vorerkrankungen, ein initial nichtdefibrillierbarer Rhythmus, Trauma als Ursache des Herz-Kreislauf-Stillstandes, die Pflegeeinrichtung als Einsatzort, das Fehlen von Zeugen und eine zunehmende Adrenalindosis mit einer abnehmenden Chance auf Überleben assoziiert. Die Überlebenschance mit guter neurologischer Erholung wird auf der anderen Seite verbessert durch Ersthelfenden-Reanimation, Zeugen und öffentliche Orte des OHCA sowie kürzere Prozesszeiten (Alarm bis Beatmung ≤ 6 min: OR =1,27; CI =1,14–1,42; *p* < 0,001; Alarm bis Defibrillation (VF/VT) ≤ 7 min: OR =1,32; CI =1,16–1,5; *p* < 0,001; Alarm bis Adrenalin (nicht VF/VT) ≤ 12 min: OR =1,28; CI =1,07–1,54; *p* < 0,01; Abb. 4).

**Abb. 5:**
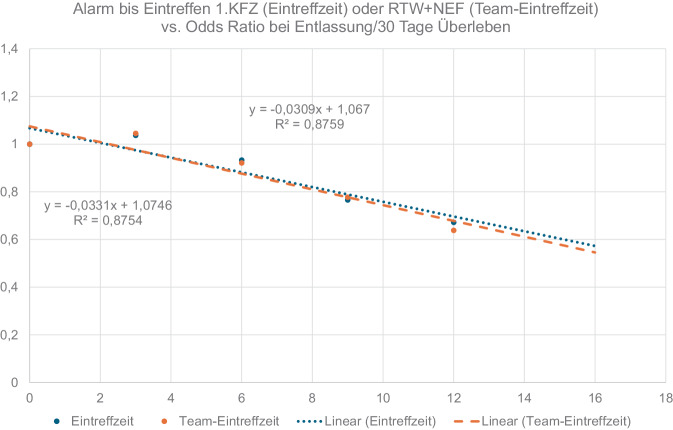
Linearer Zusammenhang von Überlebenschance (Endpunkt Entlassung/30-Tage Überleben) und Eintreffzeit sowie Team-Eintreffzeit. Die Überlebenschance verringert sich um 3,1 bzw. 3,3 % pro Minute

**Abb. 6:**
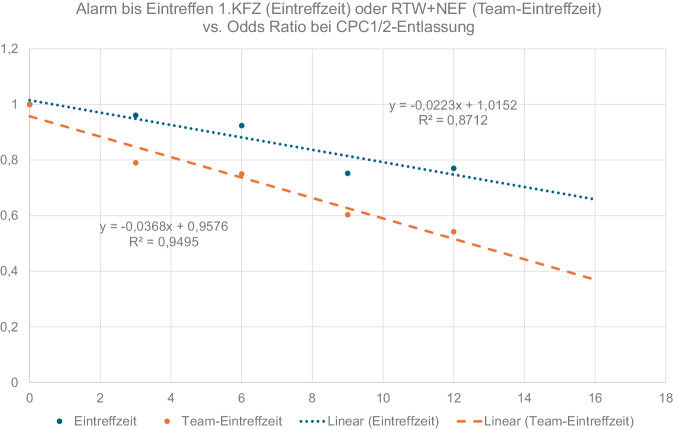
Linearer Zusammenhang von Überlebenschance (Endpunkt CPC 1‑2 bei Entlassung) und Eintreffzeit sowie Team-Eintreffzeit. Die Überlebenschance verringert sich um 2,2 % pro Minute für die Eintreffzeit und um 3,7 % für die Team-Eintreffzeit

Die grafische Auftragung der Odds Ratios zur Erreichung der Entlassung oder 30-Tage-Überlebens sowie eines Überlebens mit guter neurologischer Erholung gegen die Eintreffzeiten bzw. Team-Eintreffzeiten zeigt jeweils lineare Zusammenhänge. Die Steigungen der Regressionsgeraden quantifiziert die Verringerung der Überlebenschance pro Minute. Sie beträgt 3,1 bzw. 3,3 % pro Minute, bezogen auf den Endpunkt Entlassung aus der Klinik bzw. 30-Tage-Überleben (Abb. 5), und 2,2 bzw. 3,7 % pro Minute, bezogen auf ein Überleben mit guter neurologischer Erholung (Abb. 6). Bei einer Verzögerung der Team-Eintreffzeit um 5 min verringert sich die Chance auf ein Überleben mit guter neurologischer Erholung um 19 %.

## Diskussion

Die vorliegende Arbeit zeigt erstmalig für Bundesländer der Bundesrepublik Deutschland signifikante Unterschiede der Eintreffzeiten des ersten Rettungsmittels und der Team-Eintreffzeiten bei OHCA mit Reanimation. Verlängerte Eintreffzeiten sind mit schlechteren Prognosen assoziiert, insbesondere bezüglich der Chance auf ein Überleben mit guter neurologischer Erholung. In der Multiplikation der errechneten Chancenverschlechterung pro Minute mit den statistisch nachweisbaren Differenzen bei den Eintreffzeiten ergeben sich für betroffene Patienten relevante Unterschiede zwischen den Bundesländern in der rettungsdienstlichen Versorgung bei OHCA.

### Einordnung der Ergebnisse zu den Eintreffzeiten

Die Bundesanstalt für Straßenwesen (BAST) analysierte zuletzt im Jahr 2020/2021 die Leistungen des Rettungsdienstes durch eine räumliche Hochrechnung von Stichproben (75 von angefragten 99 Erhebungsstellen) auf der Basis der regionalstatistischen Raumtypologie. Die Eintreffzeiten aller Einsätze (Zeitraum vom Eingang der Meldung bis zum Eintreffen des ersten geeigneten Rettungsmittels) betrug in der Bundesrepublik Deutschland im Durchschnitt 8,7 min. Notfälle, die mit Sondersignal bedient werden, werden in 95% der Fälle vom ersten Fahrzeug in einer Zeit unter 16,2 min erreicht [8]. Die mit den beschriebenen Daten des GRR für die einzelnen Bundesländer errechneten Mittelwerte für Einsätze bei OHCA liegen mit 5,7 und 7,4 min deutlich unter den Ergebnissen der BAST, was durch den unterschiedlichen Startpunkt des Intervalls erklärt werden könnte (BAST: Eingang der Meldung vs. Reanimationsregister: Alarm). In einer aktuellen Untersuchung aus Europa, der EuReCa-THREE-Studie [16], konnte zum einen für Deutschland eine „Response Time“ von 8 min ermittelt, und zudem gezeigt werden, dass in einer Stichprobe von 25.711 Patienten, eine „Response time“ von mehr als 15 min die Wahrscheinlichkeit eines Überlebens bis zum Endpunkt „ROSC bei Krankenhausaufnahme“ auf etwa die Hälfte verringert (OR 0,55). Auch hier zeigte sich bei 5‑Minuten-Zeitintervallen ein annähernd linearer Zusammenhang zwischen „Response Time“ und Überlebensraten. Diese Resultate der EuReCa-Studie unterstützen die vorgestellten Ergebnisse qualitativ und quantitativ.

### Relevanz der festgestellten Unterschiede, gemessen an der Erfolgsrate bei OHCA insgesamt

Auch Unterschiede in den Eintreffzeiten von wenigen Minuten sind bei OHCA hinsichtlich des Reanimationserfolgs relevant. Die statistische Prognoseverschlechterung zeigt sich in unserer Studie am deutlichsten bei einer verlängerten Team-Eintreffzeit. Das ist ein Hinweis darauf, dass in der taktischen Einheit von RTW und Notarzt bestimmte erweiterte Maßnahmen der Reanimation effektiver umgesetzt werden können. Dies ist umso bemerkenswerter, als die Rate der Patienten mit einer guten neurologischen Erholung insgesamt nur bei etwa 7 % liegt und damit verbesserungswürdig ist [10]. Unterstützt wird diese Aussage u. a. durch Arbeiten aus Kanada und Korea, welche zeigen konnten, dass eine frühe Komplettierung des Teams besser ist als eine verspätete oder gar keine [17, 25].

### Konsequenzen für die Planung und Weiterentwicklung im Rettungsdienst sowie für die politische Diskussion

In Rettungsdienstgesetzen und anderen administrativen Regelungen der Bundesländer finden sich unterschiedliche Vorgaben und Festlegungen zur akzeptierten Zeitspanne von Hilfsfristen und Erreichungsgraden. Darüber hinaus unterscheiden sich in den einzelnen Bundesländern die Definitionen zu Beginn und Ende der Zeitspanne. Dabei scheint es unter verfassungsrechtlichen Aspekten notwendig, dass sich die konkrete Hilfsfrist „an der Korrelation von statistischer Überlebenswahrscheinlichkeit und Einsetzen der Notfallrettung, bezogen auf die in der Bevölkerung verbreiteten, lebensbedrohlichen Erkrankungen, ausrichten [muss], wobei der jeweilige Stand notfallmedizinischen Wissens maßgeblich zu berücksichtigen ist“ [8, 9].

Insgesamt stellt sich angesichts regional unterschiedlicher rettungsdienstlicher Strukturen am Beispiel des OHCA die Frage, ob Bund und Länder ihrer Verpflichtung zur Sicherstellung gleichwertiger Lebensverhältnisse hinreichend nachkommen. Forderungen zur Ausrichtung der Notfallmedizin auf eine Reihe von sog. „Tracer-Diagnosen“, worunter auch der OHCA zählt, werden im „Eckpunktepapier 2016 zur notfallmedizinischen Versorgung der Bevölkerung in der Prähospitalphase und in der Klinik“ [9] eindeutig formuliert. Unter anderem wird in dem Eckpunktepapier – bezugnehmend auf die Versorgung von Patienten mit OHCA – eine maximale Eintreffzeit von 8 min für 80 % der Patienten gefordert. In den Stadtstaaten und 3 weiteren Bundesländern wird dieses Ziel erreicht, in den anderen 10 Bundesländern wird dieses – medizinisch bewertete – Minimalziel jedoch verfehlt (Tab. 2).

Die Diskussion um Rettungsdienstplanung, Fachkräftemangel und das Ziel gleichwertiger Lebensverhältnisse erfordert einen gesamtgesellschaftlichen Konsens, welches Versorgungsniveau wir in Deutschland halten wollen, und welche Versorgungslücken in Kauf genommen werden können. Allein die Steigerung der Rettungsmitteldichte und Vorhaltezeiten werden aufgrund der Ressourcenknappheit keine Lösung sein. Um keinen Einfluss auf die politische Situation zu nehmen, verzichten die Autoren auf die konkrete Benennung der einzelnen Bundesländer. Aus unserer Sicht ist die Datenlage allein ein starker Hinweis darauf, dass es Ziel der gemeinsamen politischen und fachlichen Anstrengungen sein muss, eine einheitliche und möglichst kurze Eintreffzeit bei Patienten mit OHCA zu gewährleisten, damit für möglichst viele Betroffenen das bestmögliche medizinische Ergebnis erreicht werden kann [6, 27].

### Maßnahmen zur Verkürzung des therapiefreien Intervalls

Mit dem Ziel der bestmöglichen Versorgung der Notfallpatienten und einer gleichzeitigen effizienten Ressourcennutzung müssen alternative Konzepte zur Disposition der rettungsdienstlichen Mittel gesucht werden. Hierzu sollte z. B. das Gestufte Versorgungssystem des Kölner Rettungsdienstes [22] weiterentwickelt und bezüglich seiner Ausweitung und Adaptation auf den gesamten Rettungsdienst in Deutschland diskutiert werden. Durch die Bewertung der medizinischen Dringlichkeit, verbunden mit einem situationsgerechten Zeitfenster, und die Erweiterung des Reaktionsspektrums um weitere medizinische Ressourcen über die klassischen Notfallrettungsmittel hinaus soll eine präzisere Ressourcennutzung erreicht werden. Dazu müssen Hilfeersuchen klassifiziert und entsprechende Schutzziele definiert werden.

Hilfeersuchen, die in der Notrufabfrage auf einen OHCA schließen lassen, sollen entsprechend mit einer hohen Priorität wahrgenommen werden und zu einer Alarmierung nicht nur des nächsten RTW und NEF führen, sondern auch alle anderen – näher positionierten – Fahrzeuge des Rettungsdienstes umfassen (KTW, Kommandowagen, Transportdienste, …) [3, 19]. Zudem sollten bei Reanimationseinsätzen Strategien zu dynamischer Disposition und datenbasierter Echtzeitpriorisierung zur Anwendung kommen, wie sie in einem aktuellen Übersichtsartikel als zielführend vorgestellt wurden [2].

Zur Entlastung können dann weniger zeitkritische Notfalleinsätze durch weiter entfernte Rettungsmittel mit einer längeren Eintreffzeit beantwortet werden.

Da die rettungsdienstlichen Ressourcen das beim OHCA medizinisch erforderliche kurze therapiefreie Intervall allein nicht sicherstellen können, müssen die Leitstellen organisierte Ersthelfersysteme – wie die sog. App-Retter – fest in die Alarmierungsstichworte einbinden und deren überregionale Nutzung weiter ausgebaut werden [26, 30]. Es muss jedoch betont werden, dass bisher keine wissenschaftliche Evidenz für die Verbesserung der Überlebensraten durch diese App-Systeme gelungen ist [1, 23]. Insofern ist klar zu fordern, dass, trotz Bereitstellung von Versorgungsoptionen durch Laien und aktivierte Ersthelfer, ein frühzeitiger Beginn der professionellen Reanimationsmaßnahmen durch das Team aus Notarzt und Rettungsfachpersonal (inkl. Differenzialdiagnose, Beatmung und differenzierter medikamentöser Therapie) unverzichtbar ist und für ein gutes neurologisches Ergebnis des Patienten gewährleistet werden muss [4, 18, 28].

## Limitationen

Es muss grundsätzlich, wie bei anderen Registerstudien auch, mit einem Bias durch unvollständige und unvollzählige Dokumentation gerechnet werden. Ebenso sind Unterschiede in der Datenerfassung zwischen den Bundesländern nicht ausgeschlossen. Aufgrund der großen Fallzahlen dieser Studie kann jedoch von einer validen Datenbasis ausgegangen werden.

### Infobox Unabhängige kategoriale Variablen der logistischen Regressionsanalyse

- Eintreffzeit 1. Fahrzeug Rettungsdienst (Referenz: 0–< 3 vs. 3–< 6, 6–< 9, 9–< 12 und ≥ 12 min) oder alternativ
- Team-Eintreffzeit (beide Fahrzeuge, RTW und NEF) (Referenz: 0–< 3 vs. 3–< 6, 6–< 9, 9–< 12 und ≥ 12 min) mit Kategorien entsprechend der Referenzarbeit von Bürger et al. [6]
- Alter (Referenz: ≤ 60 vs. > 60–70, > 70–80, > 80–90 und > 90 Jahre)
- Vorerkrankungen (Referenz: ohne bekannte Vorerkrankung vs. leichte Vorerkrankung ohne Beeinträchtigung, Vorerkrankung unbekannt, Vorerkrankung mit Beeinträchtigung und schwerste Vorerkrankung oder sterbend)
- Initialer EKG-Rhythmus (Referenz: defibrillierbarer Rhythmus vs. EMD/PEA, Asystolie)
- Ursache (Referenz: kardial und unbekannt vs. Trauma, Hypoxie/Ertrinken/andere Ursache, Stroke/ICB/SAB)
- Ort (Referenz: Wohnung/anderer/unbekannt vs. Pflegeeinrichtung, Arbeitsplatz/Sport/Schule, Arztpraxis, Öffentlichkeit)
- Zeuge (Referenz: kein Zeuge vs. Ersthelfende, First Responder)
- Reanimation vor Rettungsdienst (Referenz: ohne vs. durch Ersthelfende, durch First-Responder, mit telefonischer Unterstützung),
- Adrenalindosis entsprechend CRASS [29] (Referenz: 0 mg vs. 0,1– < 2 mg, 2– < 4 mg, 4– < 6 mg, unbekannte Dosis, 6– < 8 mg, 8 mg oder mehr)
- Alarm bis Beatmung (Referenz > 6 min vs. ≤ 6 min)
- Alarm bis Defibrillation bei VF/VT (Referenz > 7 min vs. ≤ 7 min)
- Alarm bis 1. Adrenalin bei Nicht-VF/VT (Referenz > 12 min vs. ≤ 12 min)
- Alarm bis endotracheale Intubation (Referenz > 12 min vs. ≤ 12 min)

## Fazit für die Praxis

- Diese Studie zeigt relevante Unterschiede in der rettungsdienstlichen Versorgung bei OHCA zwischen den Bundesländern auf.
- Die Unterschiede von 2 bis 3 min bei den medianen Eintreffzeiten sind für ein Überleben und für ein Überleben mit guter neurologischer Erholung relevant.
- Im Hinblick auf den außerklinischen Herz-Kreislauf-Stillstand ist das Ziel der Schaffung gleichwertiger Lebensverhältnisse in den Bundesländern bisher nicht erreicht.
- Eine gesellschaftliche und politische Diskussion zur Definition der Schutzziele sowie der Frage nach einem gestuften Versorgungssystem sind daher aus der Sicht der Autoren erforderlich.

## Data Availability

Die in dieser Studie erhobenen Datensätze können auf begründete Anfrage beim Korrespondenzautor angefordert werden.
